# Supplementary figures and images for: Intrinsic Excitability in Layer IV–VI Anterior Insula to Basolateral Amygdala Projection Neurons Correlates with the Confidence of Taste Valence Encoding
Source: eNeuro. 2023 Jan 17;10(1):ENEURO.0302-22.2022. doi: 10.1523/ENEURO.0302-22.2022 (PMC9850927; doi:10.1523/ENEURO.0302-22.2022)

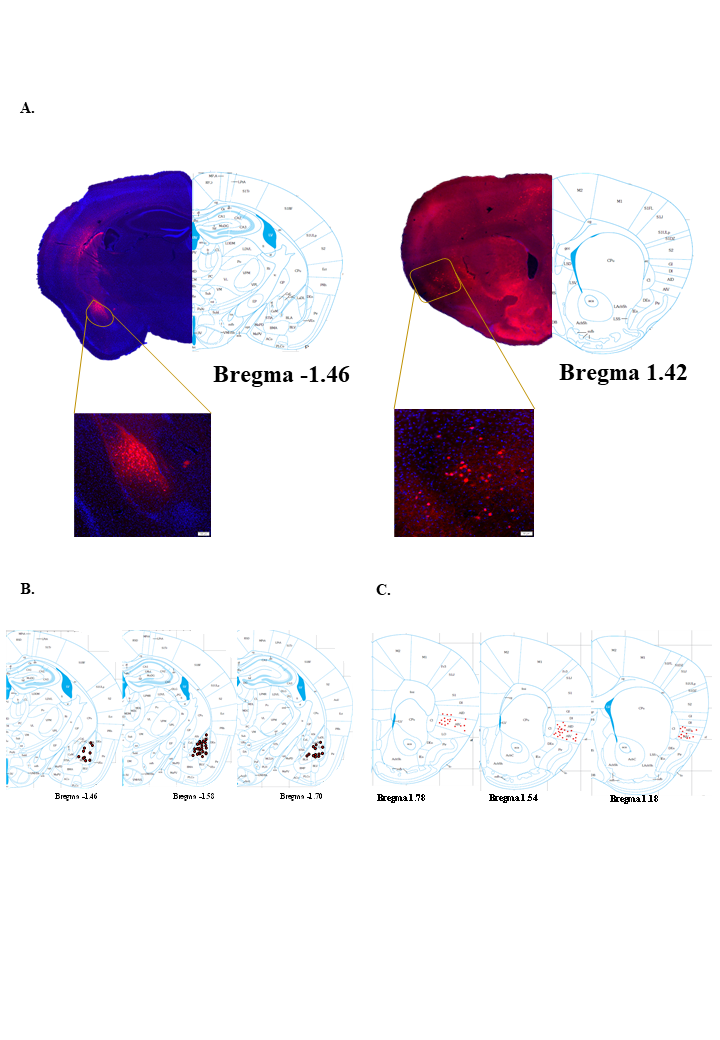

Supplement: Extended Data Figure 1-1 — Histological verification of rAAV-mCherry virus expression and locations of whole-cell patch-clamp recordings. A, A representative image showing the distribution of retrograde injections into the BLA and aIC-BLA projection neuron at aIC. B, Locations showing the retroviral injections sites in the BLA. C, Mean localization of BLA projecting neurons of the agranular aIC used for electrophysiological whole-cell recordings. Download Figure 1-1, TIF file. [file enu-eN-CFN-0302-22-s02.tif]

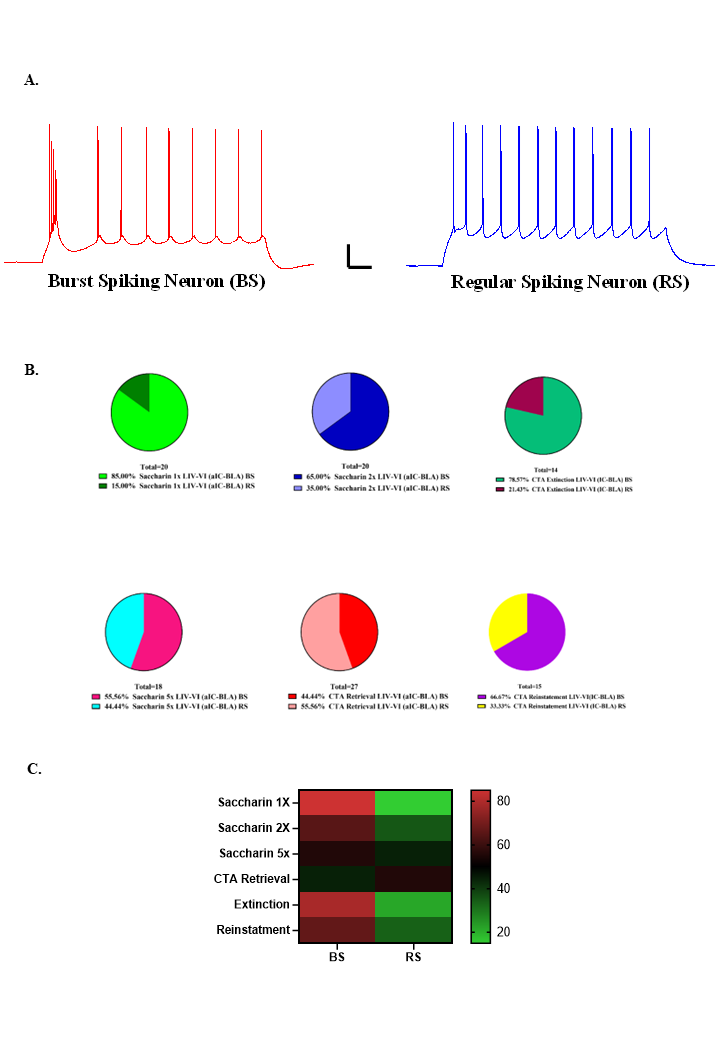

Supplement: Extended Data Figure 1-2 — The ratio of burst spiking and regular spiking LIV–VI aIC-BLA projecting neurons changes in relation to the uncertainty associated with taste experiences. A, Representative traces from Burst (BS) and Regular (RS) spiking LIV–VI aIC-BLA projecting neurons in response to rheobase current injections. The neurons showing doublets or triplets in response to rheobase current injection were considered BS. The neurons showing single spike in response to rheobase current injection considered RS. Scale bars: 20 mV and 100 ms. B, Pie charts showing the change in the ratio of BS versus RS LIV–VI aIC-BLA projection neurons, expressed as a percentage of the sampled population across the Saccharin 1x, Saccharin 2x, Saccharin 5x, CTA Retrieval, Extinction, and Reinstatement groups. C, Heat map summary of the change in the ratio of BS versus RS LIV–VI aIC-BLA projection neurons, expressed as a percentage of the sampled population across the six treatment groups. Download Figure 1-2, TIF file. [file enu-eN-CFN-0302-22-s03.tif]

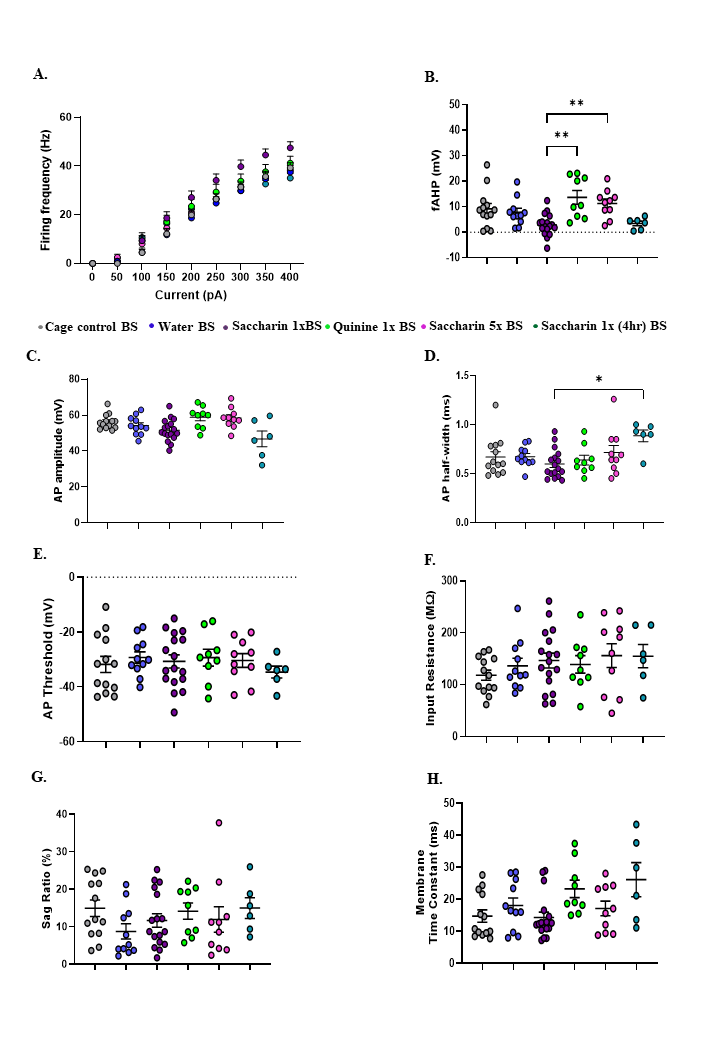

Supplement: Extended Data Figure 1-3 — Appetitive novel taste alters the intrinsic properties of burst spiking LIV–VI aIC-BLA neurons. We compared the intrinsic properties of BS and RS LIV–VI aIC-BLA neurons among the Cage Control (n = 13 cells), Water (n = 11cells), Saccharin 1x (n = 17 cells), Quinine 1x (n = 9 cells), Saccharin 5x (n = 10 cells), and Saccharin 1x (4 h, n = 6 cells). A, Excitability in BS LIV–VI aIC-BLA was not significantly different among the treatment groups. Two-way repeated measures ANOVA, Current × Treatment: p < 0.0001, Group interaction p = 0.0666. B, fAHP was significantly enhanced in Quinine 1x (13.67 ± 2.681 mV) and Saccharin 5x (11.30 ± 1.727 mV) BS neurons compared to Saccharin 1x BS neurons (2.870 ± 1.044 mV). One-way ANOVA, p = 0.0004. C, Action potential amplitude was significantly different between the groups. Cage Controls (56.27 ± 1.147 mV), Water (54.21 ± 1.572 mV), Saccharin 1x (51.64 ± 1.473 mV), Quinine 1x (58.86 ± 2.003 mV), Saccharin 5x (58.40 ± 1.812 mV), and Saccharin 1x (4 h) (46.79 ± 4.359 mV). One-way ANOVA, p = 0.0097. D, Action potential half-width in BS LIV–VI aIC-BLA neurons of the Saccharin 1x (4 h) group (0.8850 ± 0.05943ms) was increased compared to the Saccharin 1x (1 h) group, 0.5976 ± 0.03555 ms. One-way ANOVA, p = 0.0139. E, Action potential threshold was not significantly different between the groups. Cage Control (−31.83 ± 2.971 mV), Water (−29.27 ± 2.060 mV), Saccharin 1x (−30.73 ± 2.385 mV), Quinine 1x (−29.35 ± 3.071 mV), Saccharin 5x (−30.38 ± 2.493 mV), and Saccharin 1x (4 h) (−34.61 ± 2.174 mV). One-way ANOVA, p = 0.7652. F, Input resistance was similar among the different treatment groups. Cage Control (118.4 ± 9.771 MΩ), Water (136.5 ± 14.40 MΩ), Saccharin 1x (146.6 ± 14.22 MΩ), Quinine 1x (139.2 ± 16.86 MΩ), Saccharin 5x (156.1 ± 22.85 MΩ), and Saccharin 1x (4 h) (154.9 ± 22.41 MΩ). One-way ANOVA, p = 0.6304. G, SAG ratio was not significantly different between the groups. Cage Control (14.91 ± 2.195), Water (8.751 ± 2.021), Saccharin [file enu-eN-CFN-0302-22-s04.tif]

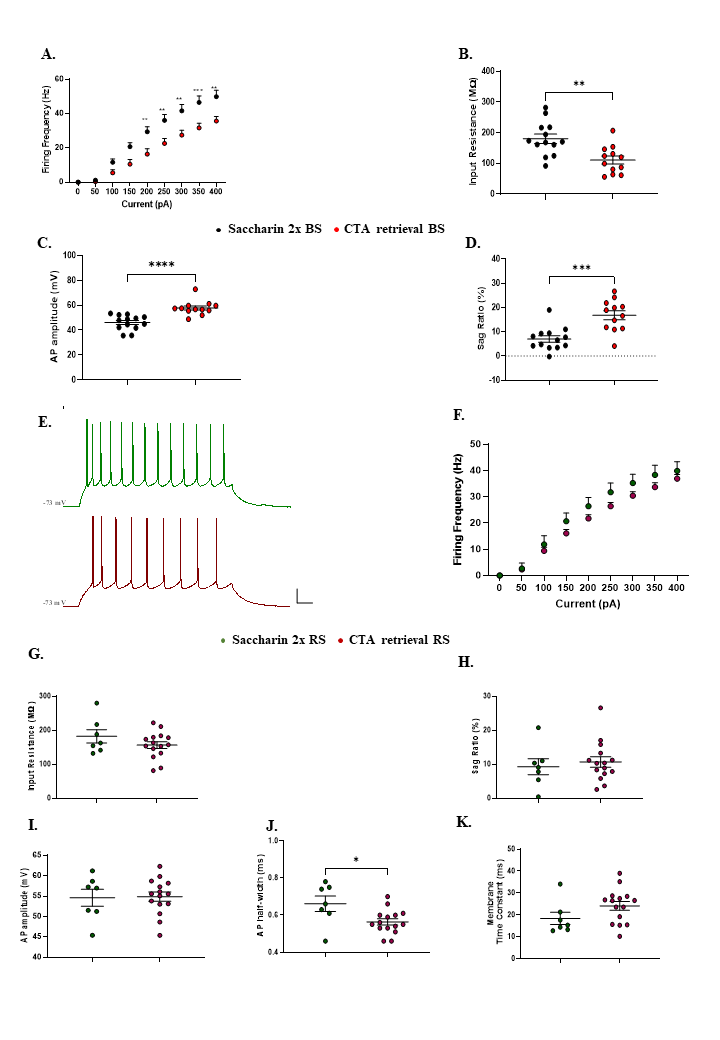

Supplement: Extended Data Figure 2-1 — Learned aversive taste memory retrieval decreases the excitability of burst spiking LIV–VI aIC-BLA neurons. We compared the intrinsic properties of BS and RS LIV–VI aIC-BLA neurons following Saccharin 2xs (BS = 13, RS = 7, cells) and CTA memory retrieval (BS = 12, RS = 15, cells). A, Excitability in BS LIV–VI aIC-BLA neurons was significantly reduced in the CTA Retrieval group compared to Saccharin 2x. Two-way repeated measures ANOVA, Current × Treatment: p < 0.0001. B, Input resistance in BS LIV–VI aIC-BLA neurons was significantly enhanced in the Saccharin 2x (180.3 ± 15.15 MΩ) compared to CTA Retrieval (110.9 ± 12.98 MΩ). Unpaired t test, p = 0.0022. C, Action potential amplitude in BS LIV–VI aIC-BLA neurons was significantly increased in the CTA Retrieval group compared to Saccharin 2x (46.18 ± 1.666 mV) and CTA Retrieval (57.87 ± 1.678 mV). Mann–Whitney test, p < 0.0001. D, SAG ratio in BS LIV–VI aIC-BLA neurons was significantly decreased in the Saccharin 2x (7.017 ± 1.317) compared to CTA Retrieval (16.8 ± 1.869). Mann–Whitney test, p = 0.0005. E, Representative traces of RS LIV–VI aIC-BLA neurons firing from the two treatments. Scale bars: 20 mV vertical and 50ms horizontal in response to 150-pA step current. F, Excitability in RS LIV–VI aIC-BLA neurons was similar in the CTA Retrieval and Saccharin 2x. Two-way repeated measures ANOVA, Current × Treatment: p = 0.0953. G, Input resistance in RS LIV–VI aIC-BLA neurons was not significantly different in between the groups. Saccharin 2x (182.6 ± 19.62 MΩ), and CTA Retrieval (156.7 ± 10.11 MΩ). Mann–Whitney test, p > 0.9999. H, SAG ratio in RS LIV–VI aIC-BLA neurons was not significantly different between the groups. Saccharin 2x (9.297 ± 2.347), and CTA Retrieval (10.71 ± 1.536). Mann–Whitney test, p = 0.5815. I, Action potential amplitude in RS LIV–VI aIC-BLA neurons was not significantly different between the groups. Saccharin 2x (54.62 ± 2.058 mV), and CTA Retrieval (54.89 ± 1.13 mV). Mann–Whitney test, p > 0 [file enu-eN-CFN-0302-22-s05.tif]

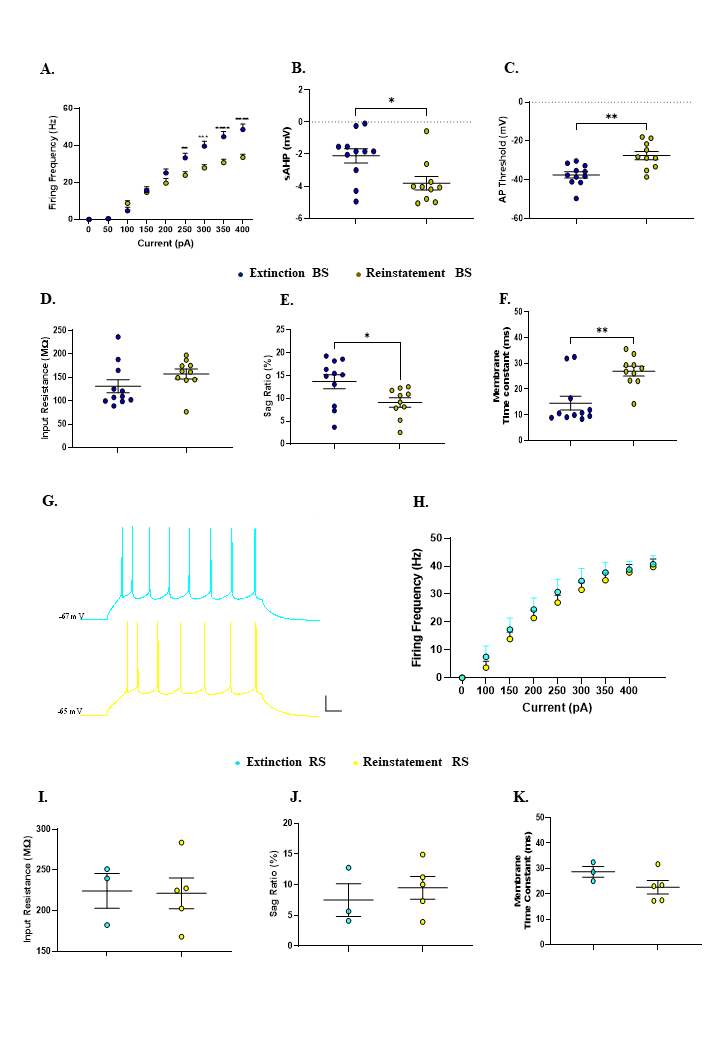

Supplement: Extended Data Figure 3-1 — Extinction of CTA enhances, excitability of burst spiking LIV–VI aIC-BLA projecting neurons We compared the intrinsic properties of BS and RS LIV–VI aIC-BLA neurons following the Extinction (BS = 11, RS = 3, cells) and Reinstatement (BS = 10, RS = 5, cells). A, Excitability in BS LIV–VI aIC-BLA was significantly enhanced in Extinction group comparing to Reinstatement. Two-way repeated measures ANOVA, Current × Treatment: p < 0.0001. B, sAHP in BS LIV–VI aIC-BLA neurons was significantly enhanced in the Extinction group (−2.104 ± 0.4466 mV) compared to Reinstatement (−3.804 ± 1.339 mV) neurons. Mann–Whitney test, p = 0.0230. C, Action potential threshold in BS LIV–VI aIC-BLA neurons was significantly reduced in the Extinction group (−37.41 ± 1.636 mV) compared to Reinstatement (−27.5 ± 2.195 mV). Unpaired t test, p = 0.0016. D, Input resistance in BS LIV–VI aIC-BLA neurons was similar in the two treatment groups. Extinction (131.1 ± 13.93 MΩ) and Reinstatement BS (157.4 ± 10.56 MΩ). Mann–Whitney test, p = 0.1321. E, SAG ratio in BS LIV–VI aIC-BLA neurons was enhanced following Extinction (13.69 ± 1.541) neurons compared to Reinstatement BS (9.124 ± 1.03). Unpaired t test, p = 0.0262. F, Membrane time constant in BS LIV–VI aIC-BLA neurons was significantly reduced in the Extinction group (14.52 ± 2.714 ms) compared to Reinstatement (26.93 ± 1.893) neurons. Mann–Whitney test, p = 0.0062. G, Representative traces of RS LIV–VI aIC-BLA firing from two treatment groups. Scale bars: 20 mV vertical and 50 ms horizontal in response to 150-pA current step. H, Excitability of RS LIV–VI aIC-BLA neurons in both treatment groups. I, Input resistance in RS LIV–VI aIC-BLA neurons was similar in the Extinction (224.2 ± 21.29 MΩ) and Reinstatement (221.2 ± 18.9 MΩ) groups. J, SAG ratio in RS LIV–VI aIC-BLA neurons was not different between the Extinction (7.515 ± 2.666) and Reinstatement (9.486 ± 1.846) groups. K, Membrane time constant in RS LIV–VI aIC-BLA neurons was not different b [file enu-eN-CFN-0302-22-s06.tif]

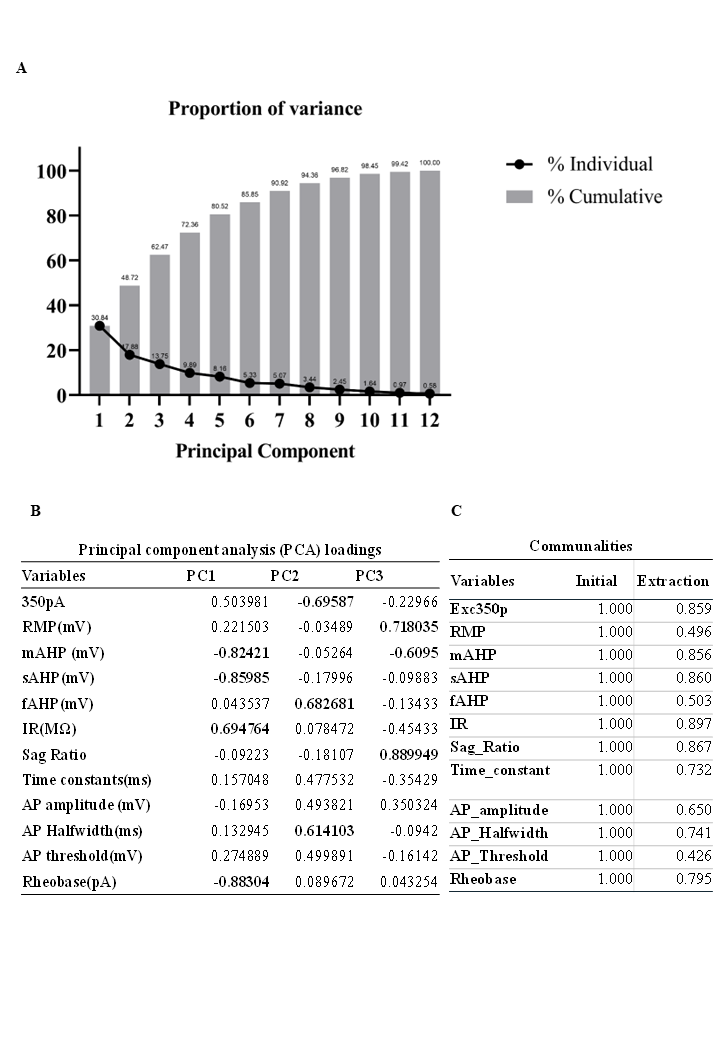

Supplement: Extended Data Figure 5-2 — PCA variable contributions and component loadings of burst-spiking and regular-spiking LIV–VI aIC-BLA projecting neurons. A, Column chart demonstrating the individual and cumulative proportion of the variance accounted by principal components following PCA of BS LIV–VI aIC-BLA projecting neurons in the two groups of treatments (Saccharin 1x, Saccharin 2x, Extinction vs CTA Retrieval, 5x Saccharin, Reinstatement). B, Table summarizing the contribution of individual variables (loadings) to the coordinate value of the principal components segregating the two groups (score). C, Communalities table, demonstrating the amount of variance in each variable that is accounted for by the extraction of principal components. Initial communalities are estimates of the variance in each variable accounted for by all components or factors (=1.00). Download Figure 5-2, TIF file. [file enu-eN-CFN-0302-22-s08.tif]

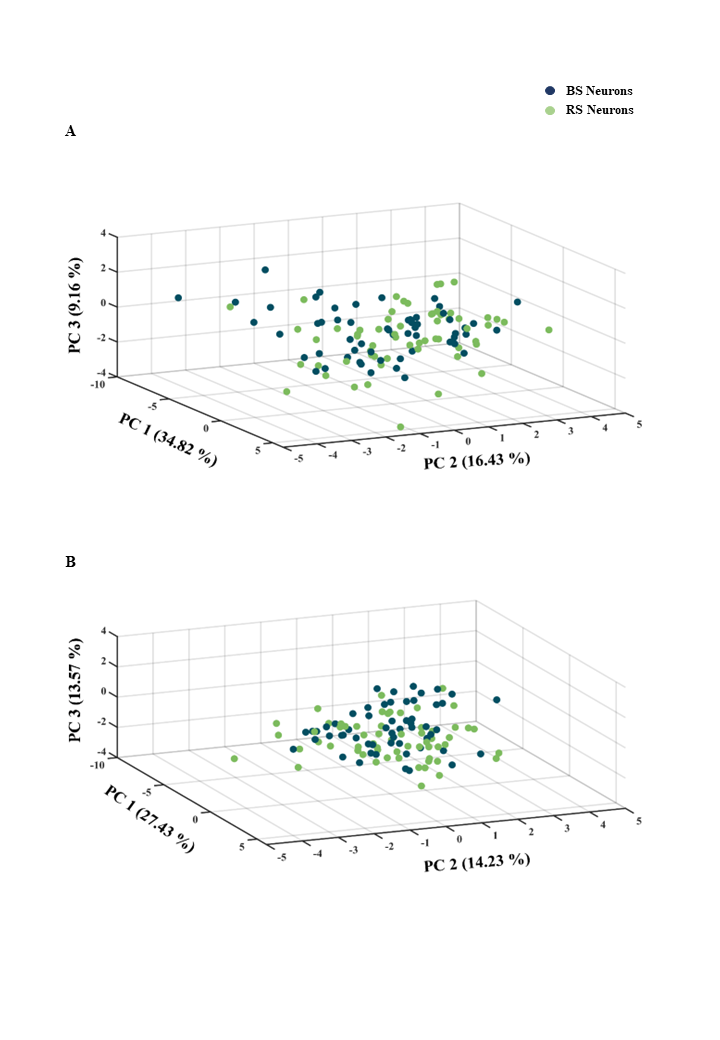

Supplement: Extended Data Figure 5-1 — PCA showing Burst versus Regular spiking LIV–VI aIC-BLA neurons all range of excitability versus 350 pA only. A, PCA of BS and RS LIV–VI aIC-BLA neurons all range of excitability (50–350 pA and all other intrinsic properties measured). Sampled population across six treatment groups (Saccharin 1x, Saccharin 2x, Saccharin 5x, CTA Retrieval, Extinction, Reinstatement). B, PCA of BS and RS LIV–VI aIC-BLA neurons excitability of 350 pA only and all other intrinsic properties measured. Sampled population across six treatment groups (Saccharin 1x, Saccharin 2x, Saccharin 5x, CTA Retrieval, Extinction, Reinstatement). Download Figure 5-1, TIF file. [file enu-eN-CFN-0302-22-s07.tif]
